# Effects of a Cognitive-Motor Training on Anticipatory Brain Functions and Sport Performance in Semi-Elite Basketball Players

**DOI:** 10.3390/brainsci12010068

**Published:** 2021-12-31

**Authors:** Stefania Lucia, Valentina Bianco, Luca Boccacci, Francesco Di Russo

**Affiliations:** 1 Department of Movement, Human and Health Sciences, University of Rome “Foro Italico”, 00135 Rome, Italy; francesco.dirusso@uniroma4.it; 2 Laboratory of Cognitive Neuroscience, Department of Languages and Literatures, Communication, Education and Society, University of Udine, 33100 Udine, Italy; biancovalentina86@gmail.com; 3 Department of Psychology, University of Rome “La Sapienza”, 00185 Rome, Italy; boccacci.1909447@studenti.uniroma1.it; 4 Santa Lucia Foundation IRCCS, 00179 Rome, Italy

**Keywords:** cognitive-motor dual-task training, ERP, task anticipation, sport performance

## Abstract

The aim of this research was to test the possible effects of cognitive–motor training (CMT) on athletes’ sport performance and cognitive functions. Namely, specific athletic tests, brain processes associated with anticipatory event-related potential (ERP) components and behavioral performance during a cognitive discrimination response task were evaluated pre- and post-training. Twenty-four young semi-professional basketball players were recruited for the study and randomly divided into an experimental (Exp) group executing the CMT training and a control (Con) group performing standard motor training. The CMT training protocol included exercises in which participants performed cognitive tasks during dribbling exercises using interactive devices which emitted visual and auditory stimuli, in which athletes’ responses were recorded. Results showed that following training, only the Exp group improved in all sport-specific tests (17%) and more than the Con group (88% vs. 60%) in response accuracy during the cognitive test. At brain level, post-training anticipatory cognitive processes associated with proactive inhibition and top-down attention in the prefrontal cortex were earlier and heightened in the Exp group. Our findings confirm previous studies on clear improved efficacy of CMT training protocols on sport performance and cognition compared to training based on motor exercises only, but extend the literature in showing that these effects might be explained by enhanced anticipatory brain processing in the prefrontal cortex. The present study also suggests that in order to achieve specific athletic goals, the brain adapts cognitive functions by means of neuroplasticity processes.

## 1. Introduction

The application of cognitive neuroscience methods in sport science is receiving increasing interest considering the crucial role it might play in improving athletic performance [1,2] by allowing innovative exercises as the cognitive-motor training (CMT). Indeed, for players of open sports such basketball, it is important to distribute attention between several cues in the environment [3] while simultaneously executing automatic motor action such as dribbling, characterizing dual-task demand [4]. CMT, by combining physical and cognitive exercises, has been proven to be more suitable than physical training alone to improve both cognitive and motor performance [5].

CMTs can be of two types depending on the exercises’ dynamics; in sequential trainings athletes perform motor and cognitive exercises in succession on the same or on different days; in simultaneous trainings (dual task), athletes perform both kinds of exercises simultaneously [6]. In this regard, there is extensive evidence that this type of CMT is more effective than sequential training [7,8,9]. While the best type of exercise to be used is still a matter of debate, the cognitive–motor dual-task (CM-DT) training, by targeting executive functioning, seems to be more beneficial for cognitive functions than sequential training and physical training alone [5].

In athletes, the goal of CMT is to augment sports performance by targeting specific skills which are transferable to competitive sports and improve general cognitive abilities [10]. The cognitive functions usually targeted by CMT include perception [11], attention, concentration, reasoning, creative thinking, memory, and decision-making [5]; however, to the best of our knowledge, proactive cognitive functions [12,13] including inhibition and anticipation remain still to be investigated. These functions are crucial for several sports but are essential in open-skill sports where athletes are continuously exposed to dynamically changing scenarios and are prompted to prepare the best action at the appropriate time [14]. This is particularly true for basketball, in that this type of sport is characterized by highly intermittent actions with changes in movement type every 1–2 s [15]. Although physical and technical/tactical attributes have been readily identified as important determinants for basketball performance [16,17], limited attention has been given to cognitive aspects [18]. Basketball’s effects on cognition have been found on attentional and inhibitory control [19,20] and on action anticipation when professional athletes have to predict the success of free shots [21]. Therefore, the ability to anticipate upcoming events and proactively control upcoming actions is fundamental in basketball and derives from a combination of sensory inputs, search strategies, effective pattern recognition and awareness of situational probabilities [22].

Starting from these sport-specific considerations, it is possible to exploit brain electrophysiological methods, and thereby to obtain information about the fast and complex dynamics of neuronal processing that occur during the execution of cognitive and motor tasks. In particular, electroencephalogram (EEG) and event-related potentials (ERPs) methods allow for the measurement of neural dynamics with millisecond precision. Thanks to high temporal resolution, ERPs have been successfully used to identify the time-course of cognitive processes ranging from task preparation to motor execution [23]. Recent literature has demonstrated that sports practice may affect anticipatory frontal ERP components, which are involved in the execution of complex cognitive sensory–motor tasks [24,25,26]. These studies used discrimination response tasks (DRT) as the Go/No-go paradigm because they require strong involvement of anticipatory cognitive functions e.g., [27]. Indeed, they focused on anticipatory pre-stimulus ERP components such as the Bereitschaftspotential (BP) and the prefrontal negativity (pN) initiating around a second before the stimulus onset.

In DRTs, it has been shown that BP amplitude predicts response times (RT), i.e., the larger the BP the faster the RT, while the pN predicts response accuracy, i.e., the larger the pN the lower the error rate [27] (for normative data). While the BP is an anticipatory readiness potential that reflects the excitability of the supplementary and cingulate motor areas and emerges prior to any voluntary act [28], the pN originating from the inferior frontal gyrus [29] has been associated with proactive cognitive functions such as top-down attention and inhibition in the prefrontal cortex [29,30,31,32]. It has been further proposed that these components represent the neural bases of an acceleration/brake (activation/inhibition) cognitive system that predicts and anticipates upcoming actions [29,31]. In DRTs one has respond as quickly and accurately as possible or abstain/inhibit an inappropriate response. Accordingly, the abovementioned studies showed that this task highly prompts both cognitive and motor preparation [33,34] making it suitable for the purpose of this research not only for the elderly or non-experts, but also for experienced athletes [25].

The main goal of the present study is to verify whether a CM-DT training using innovative interactive devices may improve sport performance in basketball players compared to physical training only. In addition, we aimed to examine the effects of the CM-DT training on behavioral performance during a cognitive DRT and the effects on anticipatory processes associated the pre-stimulus BP and pN components. For this purpose, a sport-specific CM-DT training protocol for basketball has been developed using interactive devices programmed to obtain tasks that stimulate specific cognitive functions while simultaneously training athletes physically with one of the basketball fundamentals: the dribble. Specifically, because CM-DT and DRT require common cognitive functions and especially tasks anticipation, we expect that if the CM-DT reduces the RT in the DRT, the BP amplitude should be increased. If the CM-DT improves accuracy rates, this should be reflected in enhanced pN amplitude.

## 2. Materials and Methods

### 2.1. Participants

The sample size for this study was determined the with the G*power 3.1.9.2 software [35], estimating effect size from Cohen’s f statistics. We set the expected effect size f(V) for the present mixed 2 × 2 ANOVA design at 0.6; the α level was set at 0.05, and the desired power (1 − β) at 80 (estimated sample size = 24). Therefore, 24 young male semi-elite basketball players (mean age 16.6 years; SD = 1.1) were recruited for the study. All athletes were part of the same Under-18 team of the sport society “Stella Azzurra Basketball Rome”. To quantify the expertise of the studied athletes, the Swann classification was adopted [36]. Inclusion criteria were the following: absence of any neurological and psychiatric disorders, absence of any medication during the experimental session, normal or corrected-to-normal vision, being fully right-handed (Edinburgh handedness inventory; [37] and naïve about the aim of study. Athletes were further required to be actively involved in practicing basketball and to have at least 6 years of formal training in basketball. Both parents of all participants gave their informed consent before participating in this study in accordance with the Declaration of Helsinki after approval by the local ethical committee of the University of Rome “Foro Italico”.

### 2.2. Procedure

Participants were randomly assigned to two groups of 12: the experimental (Exp) and the control (Con) group. Groups did not differ for age, education, socioeconomic status, or expertise (Swann classification 3.5 SD = 0.3 and 3.5 SD = 0.4 for Exp and Con, respectively, corresponding to semi-elite level) according to a *t*-test (t_(22)_ < 1).

**Figure 1:**
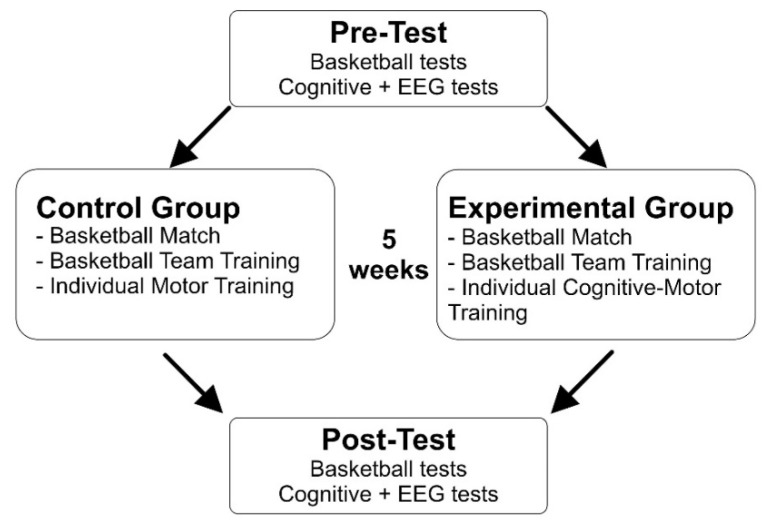
Representation of the experimental procedure.

The Con group was trained for five weeks, 7 times a week, 1 day for a basketball match (2 h) and 6 times a week for standard basketball training with group basketball training (3 h) and 2 standard individual training sessions (30 min) including physical and technical exercises only. The Exp group performed the same training schedule, but the 2 individual sessions of 30 min were done using the CMT described below. Before and after the training, all participants completed specifics tests for basketball and a cognitive task performed during EEG recording. Pre- and post-test were executed 1–2 days before and after the treatment (basketball tests 2 days before and after; cognitive and EEG tests 1 day before and after). For a representation of the experimental procedure see Figure 1.

No retention tests were made, and the were no data in terms of persistence.

#### 2.2.1. Motor Training

The standard individual training session included an activation phase, a central phase and a final phase in line with the basic principles of training where the coach focused on the fundamental of dribble with various hand, speed and direction changes ending with shots or conclusion to the basket. Exercises were arranged in routines on dribble in order to stimulate body movements with the ball in harmony and power with the aim of training the fundamental which allows you to keep and maintain the advantage over the opponent: the dribble e.g., [38]. The training was realized using one ball or two balls simultaneously. The balls were NBA and FIBA regulation size (#7), with circumferences ranging from 75 to 78 cm and weight ranging from 567 to 650 g.

#### 2.2.2. Cognitive-Motor Training (CMT)

The experimental treatment consisted of a CM-DT training requiring concurrent performance of physical and cognitive exercises, thus engaging strong inhibitory control and attention in order to disable any prepotent or distracting reaction during task performance. The task was aimed at improving both functional abilities and cognitive functions. Exercises were arranged in short routines in order to concurrently stimulate, in a coordinated manner, muscle power, static and dynamic balance and different cognitive functions. For instance, to promote inhibition of habitual responses and challenge working memory, participants performed task sequences reversing or “scattering” the learned order. Also, they were asked to learn different stimulus–response associations and then to switch between them according to the changing of external cues.

**Figure 2:**
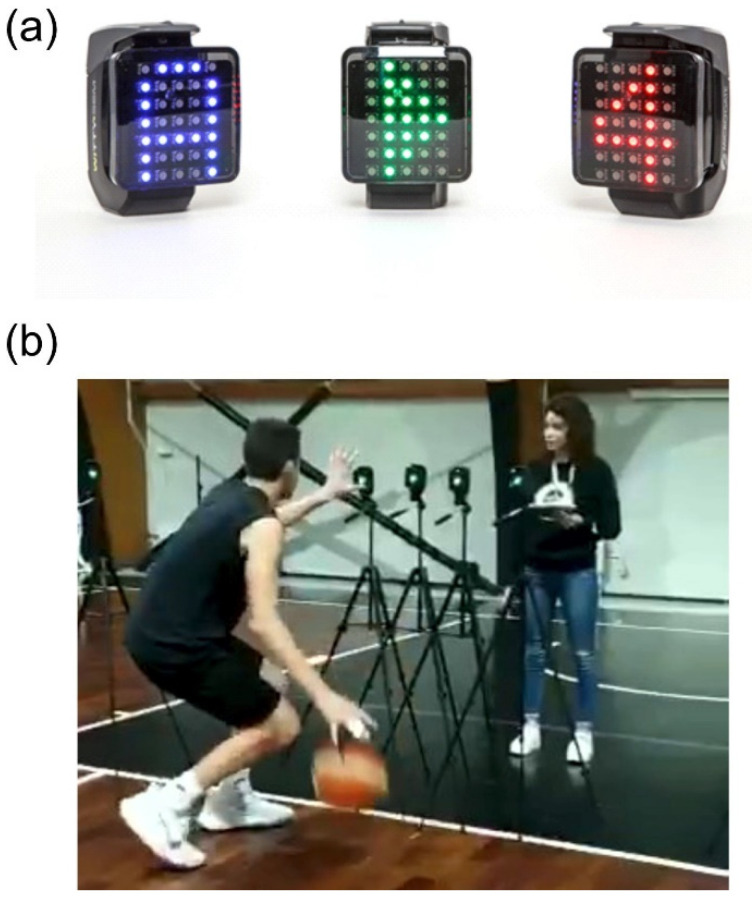
(**a**) Interactive display devices showing some of the possible visual outputs. (**b**) Video frame showing a snapshot of the CM-DT training.

The training was realized using the Witty-SEM system (Microgate, Bolzano, Italy). This system is composed of 7 × 5 led matrices that can display symbols (letters, numbers, arrows) of different colors and emit sounds that can interact with users thanks to the built-in proximity sensor. These interactive devices offer a wide range of exercises requiring cognitive functions such as attention, memory, discrimination, anticipation, and decision-making. A representation of these devices is shown in Figure 2a. During this training the Exp group was asked to perform six a CM-DT exercises requiring basketball-specific skills such as agility, precision, and control in dribbling and at the same time prompting cognitive functions including anticipation, discrimination, working-memory, and decision-making (Figure 2b). A detailed description of the single exercises is reported in the Appendix A.

#### 2.2.3. Basketball Tests

**Figure 3:**
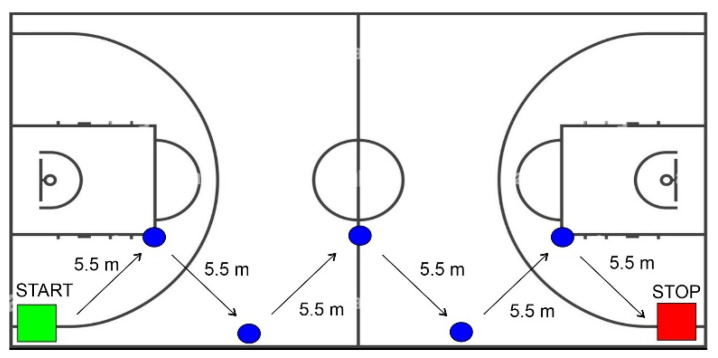
Representation on the path used in the basketball tests.

Five athletic tests based on the dribble fundamentals of basketball were used to verify the treatment effects. These tasks were based on the dribbling NBA Draft Combine tests in order to have standardized measurements. As shown in Figure 3, tests were conducted using the entire side band of the basketball court, and athletes had to dribble along a path as fast as possible while changing hand five times (every 5.5 m signaled by cones). The five tests were the following:

(1) Crossover (simple frontal hand change).
(2) Double Crossover (double frontal hand change).
(3) Between legs (hand change passing the ball between legs).
(4) Crossover + Between legs (simple frontal hand change and hand change passing the ball between legs).
(5) Between legs + Behind the back (hand change passing the ball between legs and hand change passing the ball behind the back).

For each task the test completion time was recorded and expressed in seconds.

#### 2.2.4. Cognitive Task

**Figure 4:**
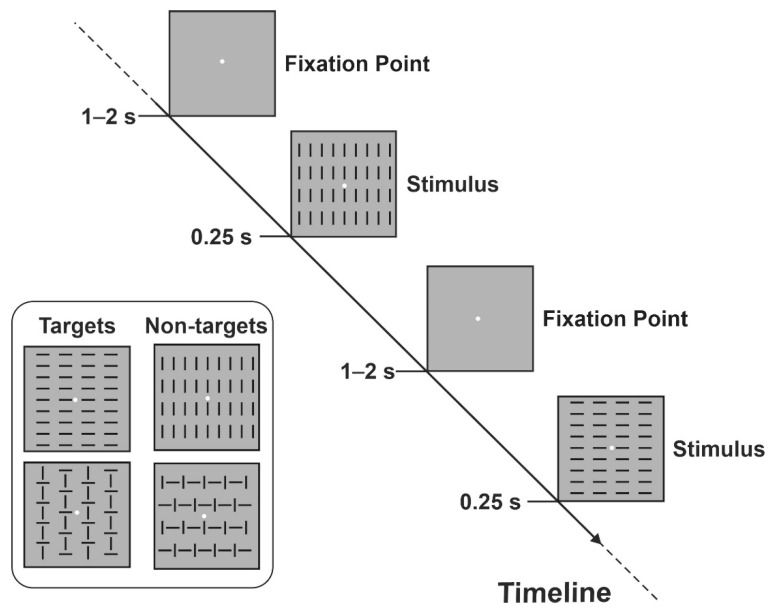
Schematic illustration of the Go/No-go task.

The cognitive task, performed while EEG traces were recorded, was done in the Cognition and Action Neuroscience Laboratory at the University of Rome “Foro Italico” and consisted of a visuo-motor DRT task, i.e., the Go/No-go paradigm. Participants were tested in a low-lit, sound-attenuated room after the EEG cap was fitted to the scalp. They were seated in front of a computer screen placed 114 cm from their eyes with the right-hand positioned palm down on a push button board. A white fixation point (diameter circle 0.15 × 0.15°) on a black background was present in the center of the screen throughout the whole experimental session. Four visual stimuli (i.e., square configurations subtending 4 × 4° and consisting of vertical and/or horizontal bars) were randomly visualized for 250 ms with equal probability (*p* = 0.25); the stimulus–onset asynchrony varied from 1 to 2 s to prevent stimulus prediction and ERP overlaps with previous and following stimuli. Participants had to press the button with the right index finger as soon as possible only when (two out of four) designed target stimuli appeared on the screen (*p* = 0.5), and to withhold the motor response when non-target stimuli appeared (*p* = 0.5); speed and precision were equally emphasized by the experimenter. Figure 4 shows a schematic representation of the stimuli and the paradigm adopted in the present Go/No-go task. The order of presentation of the four stimuli was randomized between runs. The duration of each run was 2 min with a pause interleaved. Ten runs were administered allowing us to obtain 400 trials for each stimulus category in approximately 25–30 min, depending on the individual rest time during pauses.

#### 2.2.5. Behavioral Data

Median response times (RTs) for correct trials were calculated for each participant. In order to evaluate the consistency of the response, the individual mean RT and its standard deviation (SD) were used to calculate the intra-individual coefficient of variation (ICV = SD/mean RT). Accuracy was calculated as a percentage of omissions (OM, i.e., missed responses to target stimuli), and commission errors (CE, i.e., erroneous responses to non-target stimuli).

#### 2.2.6. EEG Recording

EEG was continuously recorded with a BrainVision Recorder 1.2 using three BrainAmp^TM^ amplifiers, two of them connected to 64 active sensors ActiCap; data were processed using Analyzer 2.2 software (all by BrainProducts GmbH., Munich, Germany). Electrodes were mounted according to the 10-10 international system and referenced to average of the M1–M2 electrodes. EEG data were amplified, digitized at 250 Hz, band-pass filtered using a Butterworth zero-phase filter (0.01–40 Hz and 50 Hz notch filter; second order) and stored for offline analyses. Eye movements were monitored by electro-oculogram (EOG) recorded by a third BrainAmp amplifier (ExG type) in bipolar modality. Horizontal EOG was recorded with an electrode pair over the left and right outer canthi of the eyes, while vertical EOG (VEOG) was recorded with an electrode pair below and above the left eye. Electrode impedances were kept below 5 KΩ. Blink and vertical eye movement artifacts were automatically corrected by means of the independent component analysis (ICA [39]). Data were then submitted to automatic artifact rejection, excluding EEG with amplitudes exceeding the threshold of ±70 µV. About 2.5% of trials were rejected.

To evaluate pre-stimulus activity, EEG was segmented into 1300 ms epochs, starting 1100 ms before and ending 200 ms after stimulus onset. In line with previous studies [40,41], the baseline was applied to the first 200 ms (−1100/−900 ms) in which the signal was flat and stable. Given that the knowledge of stimulus category was unpredictable at this stage of processing, target and non-target trials were averaged together.

To select the intervals and electrodes to be considered in statistical analysis, the “collapsed localizer” method was used [42] in which a localizer ERP is obtained by collapsing (averaging) all experimental conditions. To identify the interval of analysis, the global field power (GFP) was calculated. The GFP describes the ERP spatial variability at each time point considering all scalp electrodes simultaneously, resulting in a reference-independent descriptor of the potential field [43]. The pre-stimulus interval in which the GPF was larger than 80% of its maximum value was used for further analysis. This GFP approach selected one interval from −320 ms to 0 ms in which the mean amplitude was calculated in all conditions for statistical purposes. The electrodes with an amplitude larger than 80% of the maximum value in the intervals selected by the collapsed localizer were jointed in spatial pools and considered for statistical analysis. Two foci of activity were clearly present: the medial pre-frontal activity of the pN and the medial centro-parietal activity of the BP components. The pN was then represented by a pool containing Fp1, Fpz, and Fp2 electrodes (pre-frontal pool). The BP was represented by a pool containing Cz, CPz, and Pz electrodes (centro-parietal pool).

The onset latency of the pN and the BP were also calculated using a jackknife-based procedure [44] to avoid the inaccuracies of onset latency measurements computed from a noisy single-subject ERP. Accordingly, a jackknife subsample score S_i was computed for participants by temporarily omitting participant i and calculating the pN and BP onset in the grand-average waveform computed from the remaining n − 1 participant. The onset latency was calculated as the first time point that showed a statistically significant higher amplitude than the baseline period using paired *t*-tests, n − 1 participants at pN, and BP peak electrodes (Fpz and CPz, respectively). This procedure is repeated for each subject yielding the subsample scores S_1 … S_12 for each group and condition. The subsample scores for all 12 participants were then used to estimate the standard error of the grand-average latency and for statistical analysis. We also reduced the F value Fc = F/(n − 1)^2^ as proposed by Ulrich & Miller [45] for factorial ANOVA designs to compensate the low variability of the jackknife procedure, and because the corrected ANOVA of the subsample scores is equivalent to the one of corresponding original values. Considering the higher signal-to-noise ratio of group averages than single participants, this method has been proved to be more accurate than methods of analysis using individual waveforms [44,45].

### 2.3. Statistical Analysis

For basketball tests, behavioral and EEG measures, the Shapiro-Wilk’s W test was performed to test the assumption of normality. The test showed non-significant values for all the considered measures, confirming their normal distributions. To test the assumption of homoscedasticity, the Levene’s test for equality of variance was performed, showing no violation of the sample homoscedasticity. Further, considering that distributions of RT are typically skewed to the right we calculated skewness for this data obtaining a value close to zero, thus assuming that the distribution of RT data is approximately symmetric. After this preliminary testing all measures were submitted to 2 × 2 ANOVAs with Group (Exp vs. Con) and Treatment (Pre-test vs. Post-test) as factors. For significant comparisons, effect sizes were reported in terms of partial eta squared (ηp^2^). For post-hoc comparisons the Bonferroni correction was used. Overall alpha level was fixed at 0.05. All statistical analyses were performed using the Statistica 12.0 software (StatSoft inc., Tulsa, OK, USA).

## 3. Results

### 3.1. Basketball Tests

**Figure 5:**
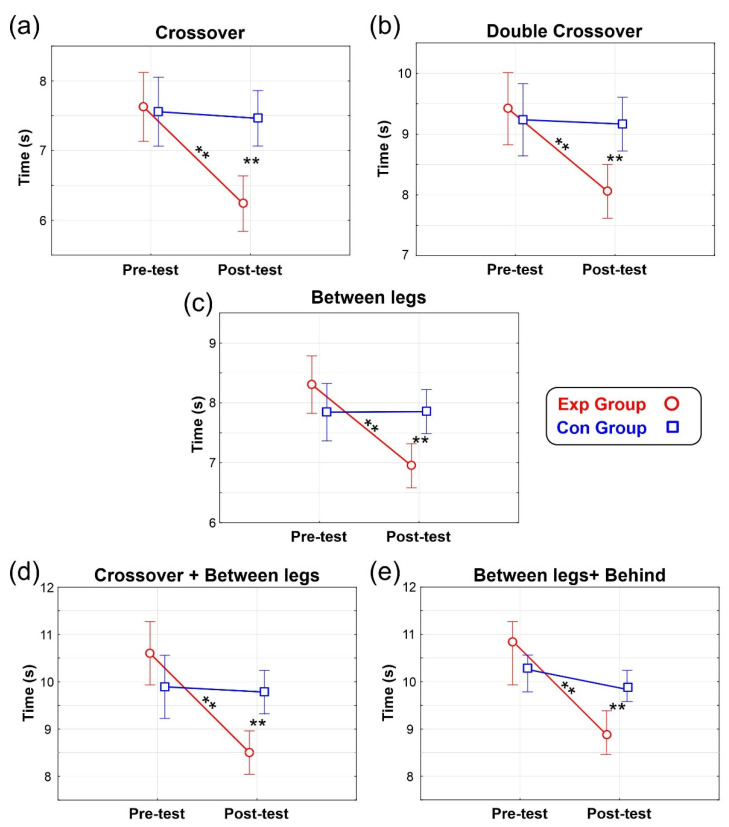
Basketball Dribbling Results. Vertical bars denote 0.95 confidence intervals. ** = *p* < 0.01. (**a**) Crossover, (**b**) Double Crossover, (**c**) Between legs, (**d**) Crossover + Between legs, (**e**) Between legs + Behind.

ANOVA on the Crossover task yielded a non-significant effect of Group (F_(1,22)_ = 3.1, *p* = 0.092, ηp^2^ = 0.12). The effect of Treatment was significant (F_(1,22)_ = 34.8, *p* < 0.001, ηp^2^ = 0.61) with shorter accomplishment time in the Post-test (6.85 s) than the Pre-test (7.59 s). The Group x Treatment interaction was also significant (F_(1,22)_ = 26.4, *p* < 0.001, ηp^2^ = 0.55). Post-hoc comparisons showed that in the Post-test the completion time of the Exp group (6.24 s) was shorter (*p* < 0.001) than the Pre-test time (7.62 s) and was also shorter (*p* < 0.001) than both the Pre-test (7.56 s) and the Post-test (7.46 s) of the Con group (Figure 5a). The other comparisons were not significant.

ANOVA on the Double Crossover task showed a non-significant effect of Group (F_(1,22)_ = 2.2, *p* = 0.155, ηp^2^ = 0.09). The effect of Treatment was significant (F_(1,22)_ = 17.91, *p* < 0.001, ηp^2^ = 0.45) with shorter accomplishment time in the Post-test (8.74 s) than the Pre-test (9.20 s). The Group x Treatment interaction was also significant (F_(1,22)_ = 14.53, *p* < 0.001, ηp^2^ = 0.40). Post-hoc comparisons showed that in the Post-test the completion time of the Exp group (8.06 s) was shorter (*p* < 0.001) than the Pre-test time (9.42 s) and was also shorter (*p* < 0.001) than both the Pre-test (9.24 s) and the Post-test (9.17 s) of the Con group (Figure 5b). The other comparisons were not significant.

ANOVA on the Between Legs task indicated a non-significant effect of Group (F_(1,22)_ = 0.6, *p* = 0.416, ηp^2^ = 0.03). The effect of Treatment was significant (F_(1,22)_ = 33.04, *p* < 0.001, ηp^2^ = 0.60) with shorter accomplishment time in the Post-test (7.40 s) than the Pre-test (8.07 s). The Group x Treatment interaction was also significant (F_(1,22)_ = 33.8, *p* < 0.001, ηp^2^ = 0.61). Post-hoc comparisons showed that in the Post-test the completion time of the Exp group (6.95 s) was shorter (*p* < 0.001) than the Pre-test time (8.30 s) and was also shorter (*p* < 0.001) than both the Pre-test (7.84 s) and the Post-test (7.85 s) of the Con group (Figure 5c). The other comparisons were not significant.

ANOVA on the combined Crossover and Between Legs task yielded a non-significant effect of Group (F_(1,22)_ = 0.8, *p* = 0.385, ηp^2^ = 0.03). The effect of Treatment was significant (F_(1,22)_ = 25.23, *p* < 0.001, ηp^2^ = 0.53) with shorter accomplishment time in the Post-test (9.14 s) than the Pre-test (10.25 s). The Group x Treatment interaction was also significant (F_(1,22)_ = 20.43, *p* < 0.001, ηp^2^ = 0.48). Post-hoc comparisons showed that in the Post-test the completion time of the Exp group (8.50 s) was shorter (*p* < 0.001) than the Pre-test time (10.60 s) and was also shorter (*p* < 0.001) than both the Pre-test (9.89 s) and the Post-test (9.78 s) of the Con group (Figure 5d). The other comparisons were not significant.

ANOVA on the combined Between Legs and Behind task showed a non-significant effect of Group (F_(1,22)_ = 1.8, *p* = 0.189, ηp^2^ = 0.08). The effect of Treatment was significant (F_(1,22)_ = 40.57, *p* < 0.001, ηp^2^ = 0.65) with shorter accomplishment time in the Post-test (9.58 s) than the Pre-test (10.65 s). The Group × Treatment interaction was also significant (F_(1,22)_ = 28.66, *p* < 0.001, ηp^2^ = 0.56). Post-hoc comparisons showed that in the Post-test the completion time of the Exp group (8.89 s) was shorter (*p* < 0.001) than the Pre-test time (10.85 s) and was also shorter (*p* < 0.008) than both the Pre-test (10.44 s) and the Post-test (10.27 s) of the Con group (Figure 5e). The other comparisons were not significant.

Correlational analysis between these tests showed that the Crossover tests significantly correlated with the Between Legs, Double Cross and Cross-Between tests. The Between Legs test significantly correlated with the Double Cross and Cross-Between tests. The other correlations were not significant. A table with all correlations is reported in Appendix B.

### 3.2. Cognitive Test: Behavioral Results

**Figure 6:**
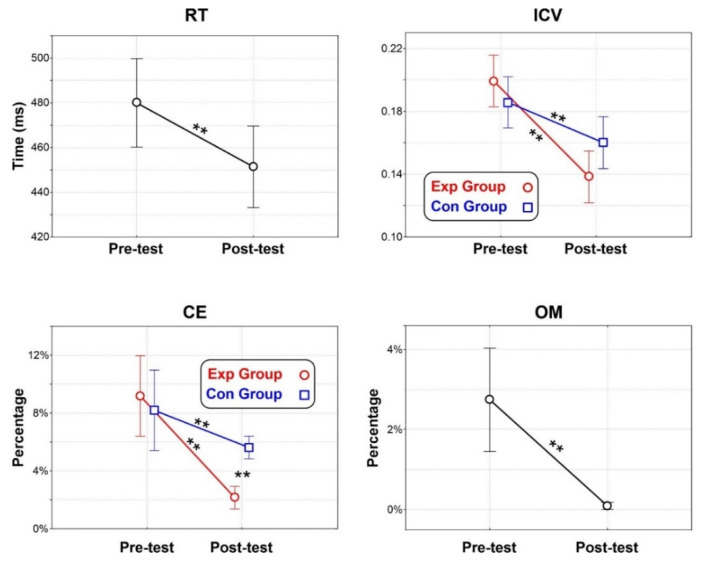
Behavioral results for response time (RT), intra-individual coefficient of variation (ICV), commission error (CE), and omission error (OM). Vertical bars denote 0.95 confidence intervals. ** = *p* < 0.01.

Behavioral data are presented in Figure 6. ANOVA on the RT yielded a significant effect of Treatment (F_(1,22)_ = 15.3, *p* < 0.001, ηp^2^ = 0.41) with shorter RT in the Post-test (451 ms SD = 9) than the Pre-test (480 ms SD = 10). The effect of Group (F_(1,22)_ = 0.4, *p* = 0.511, ηp^2^ = 0.02) and interaction (F_(1,22)_ = 0.7, *p* = 0.795, ηp^2^ < 0.01) were not significant.

ANOVA on the ICV showed a significant effect of Treatment (F_(1,22)_ = 51.8, *p* < 0.001, ηp^2^ = 0.70) with shorter ICV in the Post-test (0.149 SD = 0.025) than the Pre-test (0.192 SD = 0.028). The effect of Group (F_(1,22)_ = 0.2, *p* = 0.668, ηp^2^ < 0.01) was not significant. The Group × Treatment interaction was also significant F_(1,22)_ = 8.6, *p* = 0.008, ηp^2^ = 0.28). Post-hoc comparisons showed that in the Post-test the ICV of the Exp group (0.138 SD = 0.023) was smaller (*p* < 0.001) than the Pre-test (0.199 SD = 0.031) and was also lower (*p* < 0.001) than the Pre-test (0.186 SD = 0.027) of the Con group. In the Con group the Post-test (0.160 SD = 0.022) showed smaller ICV (*p* = 0.038) than the Pre-test. The other comparisons were not significant.

ANOVA on the CE yielded a significant effect of Treatment (F_(1,22)_ = 32.2, *p* < 0.001, ηp^2^ = 0.59) with less CE in the Post-test (3.9% SD = 0.61) than the Pre-test (8.7% SD = 1.14). The effect of Group (F_(1,22)_ = 1.2, *p* = 0.277, ηp^2^ = 0.05) was not significant. The Group × Treatment interaction was significant (F_(1,22)_ = 7.0, *p* = 0.015, ηp^2^ = 0.24). Post-hoc comparisons showed that in the Post-test the CE percentage of the Exp group (2.1% SD = 0.35) was smaller (*p* < 0.001) than the Pre-test (9.2% SD = 1.6) and was also smaller than the Pre-test (8.2 SD = 1.2, *p* < 0.001) and of the Post-test (5.7% SD = 0.7 *p* = 0.012) of the Con group. The other comparisons were not significant.

ANOVA on the OM yielded a significant effect of Treatment (F_(1,22)_ = 18.0, *p* < 0.001, ηp^2^ = 0.45) with less OM in the Post-test (0.1% SD = 0.1) than the Pre-test (2.7% SD = 0.6). The effect of Group (F_(1,22)_ = 2.1, *p* = 0.165, ηp^2^ = 0.08) and interaction (F_(1,22)_ = 2.8, *p* = 0.110, ηp^2^ = 0.11) were not significant.

Correlational analysis between these measures showed that the ICV significantly correlated with all the other measures. The OM significantly correlated with CE. The other correlations were not significant. A table with all correlations is reported in Appendix B.

### 3.3. Cognitive Test: ERP Results

**Figure 7:**
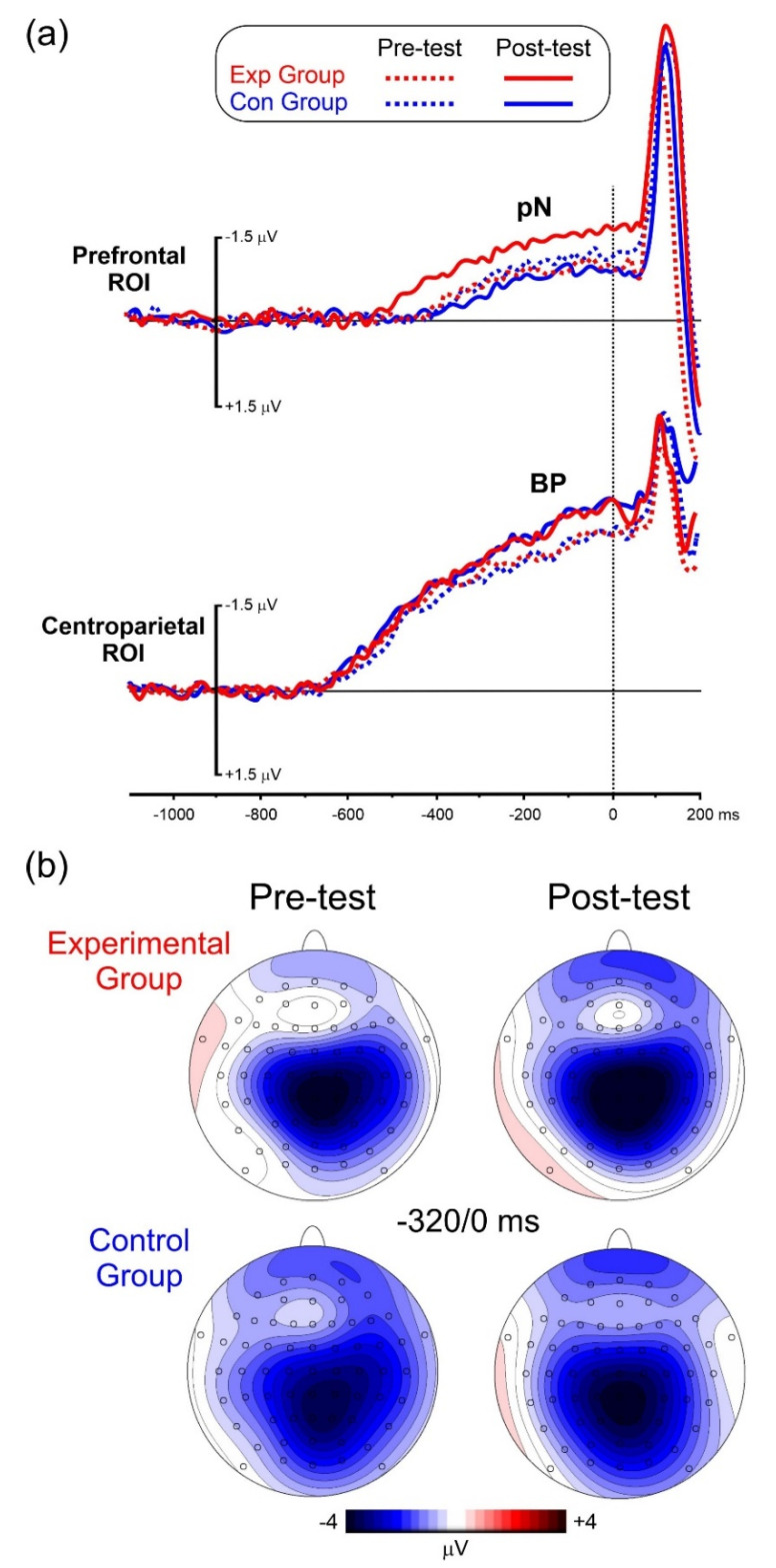
(**a**) Pre-stimulus ERP waveforms at the prefrontal and centroparietal ROIs. (**b**) Scalp topography in the −320/0 ms time window.

Figure 7a shows the pre-stimulus ERP waveforms for the four experimental conditions. Figure 7b shows the voltage and topographical distribution in the −320/0 ms interval. The BP is the first detectable activity starting from −650 ms emerging as slow-rising negativity and reaching its peak at stimulus onset on medial centroparietal sites. The pN initiated between −538 ms and −410 ms and peaked at stimulus onset on medial prefrontal sites.

ANOVA on the BP onset latency yielded a non-significant effect of Group (F_(1,22)_ = 0.7, *p* = 0.416, ηp^2^ = 0.03), Treatment (F_(1,22)_ = 0.8, *p* = 0.384, ηp^2^ = 0.04) and Interaction (F_(1,22)_ = 1.8, *p* = 0.189, ηp^2^ = 0.08).

ANOVA on the BP amplitude showed a significant Treatment effect (F_(1,22)_ = 6.3, *p* = 0.019, ηp^2^ = 0.22), with a larger Post-test amplitude (−3.12 µV SD = 0.43) than the Pre-test (−2.41 μV SD =0.31). The effects of Group (F_(1,22)_ = 2.2, *p* = 0.155, ηp^2^ = 0.09) and Interaction (F_(1,22)_ = 2.9, *p* = 0.103, ηp^2^ = 0.12) were not significant..

ANOVA on the pN onset latency showed a non-significant effect of Group (F_(1,22)_ = 0.9, *p* = 0.353, ηp^2^ = 0.04). The effect of Treatment (Fc_(1,22)_ = 8.6, *p* = 0.008, ηp^2^ = 0.28) was significant, with earlier onset in the Post-test (−545 ms SD = 62) than the Pre-test (−408 ms SD = 48). The Group x Treatment interaction was also significant (Fc_(1,22)_ = 18.7, *p* < 0.001, ηp^2^ = 0.45). Post-hoc comparisons showed that in the Post-test the pN onset of the Exp group (−538 ms SD = 0.59) was earlier (*p* < 0.001) than the Pre-test (−413 ms SD = 44) and was also earlier than the Pre-test (−410 ms SD = 0.47, *p* < 0.001) and of the Post-test (−447 ms SD = 51 *p* < 0.001) of the Con group. The other comparisons were not significant.

ANOVA on the pN amplitude showed non-significant effect of group (F_(1,22)_ = 2.4, *p* = 0.136, ηp^2^ = 0.10), treatment (F_(1,22)_ = 1.7, *p* = 0.206, ηp^2^ = 0.07), while the interaction was significant (F_(1,22)_ = 12.4, *p* = 0.003, ηp^2^ = 0.36). Post-hoc comparisons showed that in the Post-test the pN amplitude of the Exp group (−1.46 μV SD = 0.25) was larger (*p* = 0.002) than the Pre-test (−0.87 μV SD = 0.14) and was also larger than the Pre-test (−0.96 μV SD = 0.16, *p* = 0.004) and the Post-test (−0.78 μV SD = 0.12, *p* < 0.001) of the Con group. The difference between the Pre- and Pos-test in the Con Group was not significant.

## 4. Discussion

This study aimed to test the effects of cognitive–motor dual-task training on the athletic and cognitive performance of young basketball players and to investigate the underpinning effects on anticipatory brain activity using ERP measures. In particular, we focused on the BP and pN components, indexing respectively the motor and cognitive preparatory activities preceding stimulus presentation during a DRT. Results showed that the experimental treatment was the only one successful or more effective than the standard training in several of the studied variables.

Specifically, in all the basketball tests only the experimental groups improved sport performance.

Behavioral performance in the cognitive test showed reduced response times, its consistency (ICV), and omissions in both groups, but the effect on commission errors was stronger in the experimental group.

At the electrophysiological level, motor preparation for the task (BP) increased in both groups in the post-test, while the cognitive preparation (pN) was earlier and larger in the post-test in the experimental group only.

The evidence that only the cognitive–motor dual-task training improved performance in sport-specific testing in only five weeks might be related to the increased cognitive load required by the training, thus stimulating cognitive functions necessary for fast and accurate basketball dribbling, such as anticipation and selective and divided attention. Price et al. [46] have previously used a dual task to determine peak attentional demand during the free-throw process in basketball; however, authors failed to find significant differences in free-throw execution since the study was evaluating the impact of attentional disruption on performance, while in the current study, the dual task was designed to explicitly enhance both motor and cognitive performance.

The cognitive–motor dual-task training also improved accuracy performance in the cognitive test more than the standard one, likely because the dual process requested by the experimental task may especially stimulate higher cognition functions as attention facilitating response accuracy [47]. Specifically, following the intervention, the experimental group committed less commission errors than the control group. For what concerns response time and its consistency, both types of training were equally effective; this could be explained by task learning effects but also by the fact that the standard training was designed to improve decision-making speed and its consistency [48,49,50]. This is in line with the model of cognitive processes suggested by Weigel and Wollny [51] proposing that high-level athletes of team sports are especially trained to efficiently capture action-related information allowing for optimal tactical decision making. Regarding the omission rate, the values were too small post-treatment (close to zero), and a probable floor effect might have hidden possible differential effects of the two trainings, however still indicating learning effects and an excellent index of precision and readiness in young athletes.

The observed behavioral results in the cognitive test were also reflected by distinctive ERP modulations. Indeed, increased amplitude of the BP following both treatments was paralleled by faster RTs in both groups, confirming previous literature claiming a possible correlation between the anticipatory premotor activity and response speed [27].

Crucially, the pN was differently affected by the two types of training being augmented and shortened following the cognitive–motor dual-task only. This result also confirms our hypothesis about reduced commission error rate following the cognitive–motor dual-task, indicating a better attentional and inhibitory control [27]. The evidence that the pN enhancement was limited to the experimental group could be explained by the fact that this electrophysiological index of proactive cognitive control is sensitive to this kind of training and could be the neural basis of the found effects. This proactive control allows us to be active before an event occurs and is tied to specific task requests [52,53,54,55]. Considering that the pN is increased by task cognitive load [30,56], we also showed that a five-week treatment requiring high cognitive loads can modify this brain function allowing larger task accuracy even after the treatment.

The main novelty of the present study compared to previous literature using cognitive–motor dual-task training was the use of specific sports exercises together with cognitive training; indeed, other studies [57,58,59,60] used more general exercises of training of aerobic capacity, strength, balance and flexibility or walking, progressive resistance and functional balance training together with general tasks prompting executive functions, including working memory, attention or calculation ability. Here, as also in the work of Fleddermann et al. [61] with volleyball athletes, the training was more specific with respect to the study objectives and to the athletes’ characteristics.

However, some limitations have to be acknowledged: (1) in this study we only recruited male participants, but gender differences in cognitive control have been reported in previous studies [62] pointing at a more proactive and cautious approach to cognitive processing in females and more responsive and faster cognitive processing in males. Therefore, gender is an important variable to consider in future studies. (2) Given that the training design was intense and lasted for five weeks, we cannot exclude that different levels of mental and physical fatigue among groups might have modulated the pN and BP components [63] and behavioral results in the cognitive task [61]. To overcome this limitation, future studies should monitor mental and physical effort before tests. (3) We do not know how much the found effect would last after the treatment end, and a follow up would be required. (4) Considering that several of the five basketball and of the four cognitive tests were highly correlated, future studies should focus on fewer, but more independent tests. (5) Motor actions of the motor and CM-DT trainings were designed to be as comparable as possible, both focusing on dribble, but they are not identical. Indeed, both groups performed two individual dribble workouts with hand and direction changes, but while for the Con group dribbling was followed by basket conclusions, the Exp group performed the dual task and not basket conclusions.

## 5. Conclusions

Overall, the present study demonstrated that the proposed cognitive–motor training protocol was very effective in improving sport performance. This improvement was considered from a practical perspective of psychologists or coaches who want to include cognitive training in addition to technical–motor training on their team’s exercise schedule. At a neural level we showed that anticipatory prefrontal activity could be a preferential target of the cognitive–motor training. Further, we propose that the pN is not only associated with anticipatory attention and inhibition, but it is also called into play to achieve specific athletic goals, presumably because of neuroplasticity processes.

## Data Availability

Data are available from the corresponding author upon request.

## Appendix A. Cognitive-Motor Dual-Task Training Protocol

The experimental treatment protocol had a duration of 30 min in which were performed six exercises involving specific basket skills and cognitive functions at the same time. The protocol was divided in three phases (Activation, Central Phase, and Free Choice) like in normal basketball training. Each exercise lasted 1 or 3 min and was repeated two times. To respond, the players had to bring a hand close to the interactive display (brush the display).

Activation Phase (Each exercise lasted 1 min and was repeated three times).

1. Change and Touch: The player had two interactive displays positioned in front of him and he had to respond the target stimulus (colored dot) appearing in one device as fast as possible. At the same times he had to perform several dribbling hand changes typologies (i.e., crossover, between legs or between legs + behind back) depending on the target color (blue, red, green). The required basketball skills were dribbling speed and control. The required cognitive functions were discrimination, inhibition and decision-making.
2. Search: Six interactive displays were positioned in a circle around the player. While the athlete dribbled, he had to pay attention to the interactive display showing the number 7 colored in blue and to respond as quickly as possible. At the same time, he had to make dribbling hand changes of his choice. The required basketball skills were dribbling precision and agility. The required cognitive functions were attention, discrimination and decision-making.

Central Phase (Each exercise lasted 3 min and was repeated two times).

3. Find the Different: Six interactive displays were positioned in a semicircle around the player. While the player was dribbling, he had to find one target stimulus among distractors appearing simultaneously. When the player responded to the target, he had to make a dribbling hand change of his choice. The required basketball skills were dribbling precision and control. The required cognitive functions were anticipation, attention, discrimination and decision-making.
4. Found the Same: Six interactive displays were positioned in a semicircle around the player with variable heights and distances. While the player was dribbling, he had to find two identical targets among distractors that were displayed at the same time with predefined configurations. When the player responded to the two targets, he had to make a dribbling hand change of his choice. The required basketball skills were dribbling precision and control. The required cognitive functions were anticipation, attention, discrimination and decision-making.
5. Working Memory: Six interactive displays were positioned in a semicircle around the player. While dribbling, the payer had to order six numbers appearing simultaneously and briefly. Once done, he had to make a dribbling hand change of his choice. The required basketball skill was dribbling control. The required cognitive functions were attention, working-memory and decision-making.

Final Phase (The exercise was repeated two times).

6. Free Choice. The player had to repeat an exercise of his choice trying to improve the previous performance.

In all the exercises target frequency and duration was adaptively changed automatically depending on the response time. The height and distance of interactive displays was adapted to the player height using tripods.

## Appendix B

**Table A1:** Correlations between basketball tests results in both groups and sessions (*n* = 48). Values in red denote significant correlations at *p* < 0.05.

	Crossover	Between Legs	Double Cross	Cross-Between
Crossover				
Between Legs	0.70			
Double Cross	0.67	0.62		
Cross-Between	0.60	0.67	0.30	
Between-Behind	0.11	0.12	−0.24	−0.07

**Table A2:** Correlations between behavioral cognitive test results in both groups and sessions (*n* = 48). Values in red denote significant correlations at *p* < 0.05.

	RT	CE	OE
RT			
CE	0.06		
OE	0.07	0.71	
ICV	0.41	0.67	0.61
